# Learners’ characteristics and the mastery of digital education during the COVID-19 pandemic in students of a medical faculty in Germany

**DOI:** 10.1186/s12909-023-04012-x

**Published:** 2023-02-02

**Authors:** Julia Roick, Paul Poethke, Matthias Richter

**Affiliations:** Martin Luther University Halle-Wittenberg, Institute of Medical Sociology, Magdeburger Str. 8, 06112 Halle (Saale), Germany

**Keywords:** Medical education, COVID-19, Digital education, Medical faculty students, learning behavior

## Abstract

**Background:**

In response to the spread of the coronavirus, educational institutions have been closed and digital education has become a new teaching method to ensure the continuity of medical education. Since this format was a new form of learning for students at medical faculties in Germany, little is known about the perception of it and the factors that contribute to successful mastery. The current study aimed to analyze students’ learning experiences during the first online semester and to identify associations between learners’ characteristics and enjoyment, mastery experiences, as well as the perceived stress level.

**Methods:**

In this cross-sectional study, students of a medical faculty from Germany answered an online questionnaire including information about perceptions towards digital education and learners’ characteristics (study skills and dispositions). Data were analyzed using multivariate linear regression analysis.

**Results:**

In total, 383 students responded to the online survey. A majority of students felt at least somewhat worse about their studies compared to before the pandemic. Success of study tasks was related to preferences for cooperative learning (B = − 0.063, *p* < .001) and success of study organization was associated to the use of metacognitive learning strategies (B = 0.019, *p* = .04). Enjoyment of studying in times of digital education was positively related to the use of metacognitive strategies (B = 0.049, *p* = .04) and self-efficacy (B = 0.111, *p* = .02). The perceived stress was influenced by cognitive strategies (B = 0.401, *p* = .02) and test anxiety (B = 0.466, *p* < .001).

**Conclusions:**

Although students perceive digital teaching as a good alternative for big courses, those with low self-efficacy beliefs and low self-regulation have problems in coping with the demands of this learning format and need further support.

## Introduction

The rapid spread of the coronavirus SARS-CoV-2 led to numerous challenges around the world and adaptions in various areas of social life were necessary. People were forced to restructure their daily routine both in work by an increase in home office and in private life by social distancing and isolation. Universities were also strongly affected by the COVID-19 pandemic, with teaching having to be restructured from in-class learning to online lectures and seminars [1, 2]. These changes were particularly serious for students at medical faculties in Germany, as the previous course of study consisted exclusively of in-person courses. While asynchronous learning and online courses were already present in other subjects or in distance universities, this concept was completely new at medical faculties in Germany. In order to find out whether digital education will be a useful supplement or extension to the existing teaching concept in medicine, it is important to know how students perceived online teaching and how the new format affected their learning success. Furthermore, it is of great interest to identify and strengthen those factors that are associated with learning and stress management in digital education since blended learning concepts will become more and more important due to the change in teaching during the pandemic. Especially since distance and classroom teaching are not comparable in their outcomes [3], these data are of particular interest.

The study of medicine is associated with various stressors. Students who study a medical subject have to cope with constant achievement pressure, large workloads, a high number of assessments, and difficulties resulting from clinical demands. All these factors lead to a high level of distress. According to a meta-analysis, nearly one third of medical students suffers from depressive symptoms [4]. Therefore, it is important to evaluate how stressful online teaching is perceived among students at medical faculties and what factors promote successful coping and enjoyment of studying. In their Competency Model for Studying, Learning and Performing Under Stress (SLPS), de la Fuente et al. [5] propose a theoretical foundation for explaining learning outcomes in stressful situations. According to SLPA, presage variables refer to the learning situation or context, in the case of this study the special situation of the pandemic. These variables interact with process variables and the outcome. Process variables include characteristics of students (e.g., study skills, attitudes, and habits) which influence cognitive and emotional aspects of learning [6]. For the present study, we focused on study skills (cognitive and metacognitive learning strategies and preferences for cooperative learning) and dispositions (test anxiety and self-efficacy). Both, presage and process variables influence stress perceptions according to SLPA. In our study, we broaden the scope and in addition to stress levels we also considered mastery experiences and enjoyment as outcomes.

For the context of in-class learning in higher education, research yielded multiple evidence that learner’ characteristics, such as self-efficacy beliefs, self-regulated learning, or achievement goals are associated with academic performance [7–11]. The higher a student’s self-efficacy, the better their self-regulation strategies, and, consequently, their achievement [12]. Especially cognitive strategies that refer to mental abilities like rehearsal and establishing associations, and metacognitive control strategies that monitor the learning process constitute predictors of academic achievement [8, 9, 13, 14]. Further, cooperative learning and social support as indicators for the learning atmosphere have positive effects on learners’ feelings and in turn promote academic performance [15]. Text anxiety, as a major determinant of performance in higher education [9], has been found to negatively influence academic success [7].

If and to what extent those relationships can also be found in online learning settings in students at medical faculties remains unclear so far. In response, the current study examined perceptions of digital education in students from a medical faculty during the COVID-19 pandemic and investigated learners’ characteristics, more specifically study skills and dispositions, as predictors of enjoyment and mastery experiences in online seminars as well as the perceived stress level of the students compared to their studies before the pandemic.

## Methods

### Design and data collection

Participants of the cross-sectional study were students from the Medical Faculty of the Martin Luther University Halle-Wittenberg, Germany. Data collection was done between July and September 2020 using an online survey, which was distributed via the student email list of the student union of the medical faculty. Students received a link to take part in the survey. On the website of the study, students were thoroughly informed about the voluntary nature of their participation, which means that responses to questions were not obligatory. All students were asked to give informed consent before the beginning of the survey. Participants had the opportunity to take part in a raffle for various vouchers (e.g. Amazon, book store) at the end of the survey. If they agreed to take part, contact information (email address), was collected to be able to contact them in case of a win. If they did not want to participate in the raffle, no identifying information was required to protect participants’ anonymity. The study was conducted in accordance with the Declaration of Helsinki and its latter amendments. Ethical approval was obtained from the Ethics Committee of the Medical Faculty at Martin Luther University Halle-Wittenberg (reference number 2022–054).

### Measures

We collected sociodemographic information via standardized questions as well as the following measures:

#### Perceptions of digital education

Perceptions towards digital education were evaluated through four single questions (e.g. “Compared to before the Corona virus, I am doing as follows in terms of my studies.”). Answers could be given on a five-point Likert scales ranging from “better” to “worse” or “exactly right” to “not true at all”.

#### Academic self-efficacy

Academic self-efficacy was assessed using the German Academic Self-Efficacy Scale developed by Jerusalem and Satow [16]. This eight item self-report questionnaire assesses students’ subjective beliefs regarding his or her ability to deal with high demands related to academic performance (e.g., “Even if a lecturer doubts my abilities, I am sure that I can achieve a good performance.”). Reponses can be given on a four-point Likert scale ranging from “not at all” to “a great deal”. The scale ranges from 8 to 32 whereas higher scores imply more self-efficacy beliefs. This questionnaire has been used several times in studies to measure self-efficacy in the academic setting and the overall reliability and validity of the scale have been found to be good [7, 16, 17]. In the current study, reliability was good (Cronbach’s alpha =0.76).

#### Test anxiety

Test anxiety as a situation-specific personality disposition was measured by the Test Anxiety Questionnaire [18]. This instrument assesses the four facets of test anxiety (emotionality, worry, interference, and lack of confidence) by means of 20 items (e.g., “I think about what happens if I do poorly.”). Answers can be given on a four-point Likert scale ranging from “hardly ever” to “nearly always”. The total scale ranges from 20 to 80, with higher scores implying higher test anxiety. Internal consistencies and retest reliabilities of the questionnaire have proven to be very good [19, 20]. Reliability was also very good in the current sample (Cronbach’s alpha =0.91).

#### Stress level

We administered an analogue scale ranging from 0 to 100 to assess the actual stress level of the students.

#### Enjoyment

Enjoyment was investigated through three self-created questions. Students were asked to indicate to what extent they agree with the statements whether studying is fun, exciting or they enjoy learning (e.g., “Right now I’m really enjoying learning and working in my studies.”). Answers could be given on a five-point Likert scale ranging from “exactly right” to “not true at all” whereas higher scores imply more enjoyment. In the current study, reliability of the three item scale was very good (Cronbach’s alpha =0.89).

#### Mastery experiences

Mastery experiences were measured by means of a single item each. Students were asked to indicate their success in study tasks and in study organization in comparison to before the pandemic (e.g., “Compared to before the Corona virus, how well do you succeed in the tasks for your study?”). Answers were given on a five-point Likert scale ranging from “better” to “worse”. Higher scores in the items imply more success in study tasks and organization.

#### Learning strategies

We assessed learning strategies using the Questionnaire for Measuring Learning Strategies in Higher Education (LIST) [13]. Cognitive strategies were measured with the eight-item subscale *establishing associations* (e.g., “In my thoughts, I try to combine the learned things with the information I already know about it.”). Metacognitive strategies were evaluated with the six-item subscale goal setting and planning (e.g., “I think about how I want to learn, before I start.”). For both subscales, responses could be given on a six-point Likert scale ranging from “not at all” to “a great deal”. Reliability was good for both subscales (cognitive strategies: Cronbach’s alpha =0.83; metacognitive strategies: Cronbach’s alpha =0.84). Those strategies constitute important predictors for academic performance [21].

#### Cooperative learning

To capture preferences for cooperative learning, we used the scale of Marsh et al. [22] used in the PISA studies and originally created by Owens & Straton [23]. This five-item scale assesses whether students enjoy learning in cooperative situations (e.g., “I like to work with other students.”). Answers can be given on a four-point Likert scale ranging from “disagree” to “agree”. In the current sample, reliability was good (Cronbach’s alpha =0.84).

### Statistical analyses

Descriptive statistics were computed for all study variables. Bivariate correlations between the variables were conducted using Pearson correlation coefficient. Multivariate linear regression analyses were conducted to examine learner’s characteristics (study skills: cooperative learning, goal setting and planning, establishing associations; dispositions: test anxiety, self-efficacy) as predictors for mastery experiences (success in study organization, and in study tasks), enjoyment, and stress-level. In a first step, study skills were added to the models (model 1) and in a second step, dispositions were additionally added (model 2). For these analyses, an increase of 10 observations in the sample size is necessary for each additional independent variable [24]. Hence, for our analyses, a minimum of 50 observations is necessary which we have fulfilled with our sample size. Missing values were replaced using multiple imputation, which explicitly considers the uncertainty associated with the estimation of the missing values in the test statistics [25]. Data analysis was done using IMB SPSS Version 25.

## Results

### Sample characteristics

Initially, 528 students (approx. 50%) gave consent to take part, though 34 students have not answered any of the questions. Thus, 494 students were included in the study. Of those, 383 (78%) answered the questions about their perceptions towards digital education. Participants were between the ages of 18 and 45 (16 did not report their age) covering different study programs (Table 1). None of the participants was under 18 years. The majority of the students were female (72.1%) and reported German as their nationality (94.8%).

**Table 1:** Characteristics of medical student respondents. (*n* = 383)

Characteristics	All n (%)
Age years, mean (SD)	24.06 (4.15)
Gender	
Male	104 (27.2)
Female	276 (72.1)
Non-binary	3 (0.8)
Study program	
Human medicine	318 (83.0)
Dental medicine	44 (11.5)
Evidence-based nursing	10 (2.6)
Health and nursing sciences	11 (2.9)
Nationality	
German	363 (94.8)
Other	20 (5.2)
Year of study	
1	58 (15.2)
2	86 (22.4)
3	63 (16.4)
4	75 (19.5)
5	56 (14.6)
6	45 (11.7)

### Perceptions of digital education

**Fig. 1:**
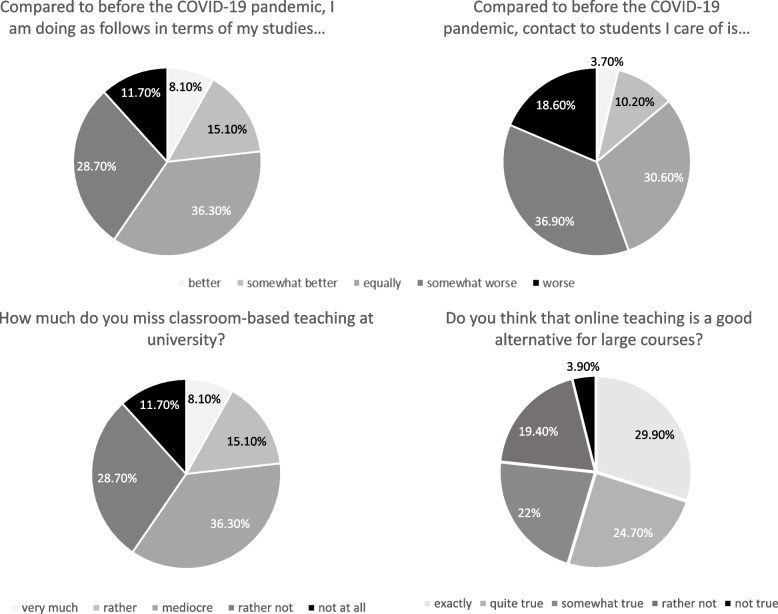
Perceptions of digital education

About 40% of the student’s report that they felt at least somewhat worse or worse about their studies compared to before the COVID-19 pandemic (Fig. 1). Over three quarter is missing classroom-based teaching moderate to very much. However, also more than 75% agree with the statement that online teaching is a good alternative for big courses. More than half of the student’s report that contact with students they care about has worsened compared to before the pandemic.

### Descriptive statistics and bivariate correlations

Table 2 provides descriptive statistics and internal consistencies of the study variables. Overall, students report a relatively high stress level (*M* = 67.20), a medium level of enjoying studying (M = 8.82) as well as medium levels of success in study tasks (M = 3.11), and success in study organization (M = 3.12). In line with theoretical assumptions, a high stress level is associated with higher test anxiety (*r* = .33, *p* < .001), and lower self-efficacy (*r* = −.25, *p* < .001). Further, enjoyment is negatively related to test anxiety (*r* = −.22, *p* < .001) and positively related to self-efficacy (*r* = .23, *p* < .001). As assumed, enjoyment (*r* = .11, *p* = .04) and success in study organization (*r* = .10, *p* = .05) are higher, the more metacognitive learning strategies are used. Contrary to previous study results, the use of cognitive learning strategies is positively related to the perceived stress level (*r* = .11, *p* = .04) implying that a higher use of this strategies results in increased stress. Finally, success in study tasks is negatively related to preferences for cooperative learning (*r* = −.20, *p* < .001), implying that students who prefer to learn with others are less successful in their study tasks during digital education.

**Table 2:** Descriptive statistics, alphacoefficients and bivariate intercorrelations among the study variables

Measure		2.	3.	4.	5.	6.	7.	8.	9.	M	SD	Range
1. Stress level^a^	–	−.15^**^	−.13^**^	−.30^**^	.33^**^	−.25^**^	.08	.11^*^	.04	67.20	23.09	0–100
2. Success in study tasks^a^		–	.70^**^	.43^**^	−.06	.07	.08	.03	−.20^**^	3.11	0.98	1–5
3. Success in study organization^a^			–	.34^**^	−.08	.05	.10^*^	.03	−.07	3.12	1.12	1–5
4. Enjoyment				.89	−.22^**^	.23^**^	.11^*^	.07	.03	8.82	2.92	3–15
5. Test anxiety					.91	−.60^**^	.01	−.03	−.14^**^	46.11	10.91	20–80
6. Self-efficacy						.76	.05	.19^**^	−.01	21.51	4.08	8–32
7. Metacognitive strategies							.84	.20^**^	.05	16.25	6.36	6–36
8. Cognitive strategies								.83	.13^*^	35.83	6.69	8–48
9. Cooperative learning									.84	14.60	3.46	5–20

Bivariate Pearson correlations are presented as standardized coefficients; Cronbach’s alpha coefficients for each construct are presented in the diagonal. ^a^Single Item. *p ≤ .05; **p ≤ .01

### Associations between learners’ characteristics and mastery experiences, enjoyment, and stress level

Since there were no associations between the outcomes variables and age (*p* = 0.18 to 0.44) or sex (*p* = 0.28 to 0.94), both variables were not entered as covariates in the multivariate models. Because of the negative relationship between semester and stress level (*r* = −.34, *p* < .01), implying that students with advanced studies were less stressed, this variable was added as a covariate in multivariate analysis on predictors of stress experiences.

In the multivariate models (models 2, Table 3), the following results were found when controlling for study skills and dispositions: 1) Success in study tasks was lower when students had preferences for cooperative learning (B = − 0.063, *p* < .001). 2) Success in study organization was higher, the more students used metacognitive learning strategies (B = 0.019, *p* = .04). 3) A greater use of metacognitive learning strategies (B = 0.049, *p* = .04) and higher self-efficacy (B = 0.111, *p* = .02) increased enjoyment of studying. 4) A higher use of cognitive learning strategies (B = 0.401, *p* = .02) and higher test anxiety (B = 0.466, *p* < .001) increased the perceived stress during digital education.

**Table 3:** Learner’s characteristics as predictors of mastery experiences, enjoyment, and stress level

Outcomes	Predictors	Model 1		Model 2	
		B	CI	B	CI
Success of study tasks	Metacognitive strategies	0.013	− 0.003; 0.028	0.013	−0.002; 0.029
	Cognitive strategies	0.005	−0.020; 0.010	0.005	−0.020; 0.010
	Cooperative learning	−0.058^***^	0.030; 0.086	−0.063^***^	0.034; 0.091
	Self-efficacy			−0.002	−0.028; 0.033
	Test anxiety			−0.010	−0.002; 0.021
	R^2^	.05		.07	
Success of study organization	Metacognitive strategies	0.018^*^	0.000; 0.036	0.019^*^	0.001; 0.037
	Cognitive strategies	0.003	−0.020; 0.015	0.003	−0.021; 0.015
	Cooperative learning	−0.026	−0.006; 0.059	− 0.031	−0.002; 0.064
	Self-efficacy			−0.006	−0.029; 0.042
	Test anxiety			−0.011	−0.003; 0.024
	R^2^	.02		.06	
Enjoyment	Metacognitive strategies	0.049^*^	0.001; 0.097	0.049^*^	0.002; 0.096
	Cognitive strategies	0.020	−0.066; 0.026	0.007	−0.052; 0.039
	Cooperative learning	0.016	−0.101; 0.070	0.007	−0.092; 0.078
	Self-efficacy			0.111^*^	−0.202; − 0.020
	Test anxiety			−0.032	−0.002; 0.066
	R^2^	.02		.10	
Stress level^a^	Metacognitive strategies	0.177	−0.525; 0.172	0.165	−0.498; 0.169
	Cognitive strategies	0.330^*^	−0.003; 0.664	0.401^*^	0.073; 0.729
	Cooperative learning	−0.176	−0.817; 0.464	0.051	−0.578; 0.680
	Self-efficacy			−0.532	−1.202; 0.138
	Test anxiety			0.466^***^	0.216; 0.716
	R^2^	.13		.23	

*p ≤ .05; **p ≤ .01; ***p ≤ .001. ^a^Model controlled for semester

## Discussion

The current study investigated the role of learners’ characteristics, in particular study skills and dispositions, and their relation to enjoyment, the perceived stress, and mastery experiences in the setting of digital education during the COVID-19 pandemic in German students of a medical faculty.

Compared to before the COVID-19 pandemic, a large proportion of students felt at least somewhat worse about their studies and missed classroom-based teaching as well as contact to peers. Also in other countries, a large majority of medical students reported that they had more difficulties with online learning than in normal classes [26, 27]. This may be due to the fact that online teaching is seldom used at medical faculties and students are not used to it. However, some students seem to cope better with the new format than others. Especially students with high self-efficacy were able to handle digital education during the pandemic. This is in line with theoretical assumptions, because students with high self-efficacy beliefs have the conviction to successful reach academic goals and thus experience less stress [28]. Exactly the opposite is true for test anxiety, leading to an increased perception of stress. Previous studies among students from other disciplines also found these relationships [7]. Nevertheless, in our multivariate model, only test anxiety was associated with stress, implying that this seems to be a stronger predictor for the perception of stress than self-efficacy beliefs. Regarding academic performance, it was found exactly the opposite, i.e. self-efficacy better predicts performance than anxiety [29, 30].

Surprisingly, the use of cognitive learning strategies was positively associated with stress in our sample. This means that the more students tried to establish associations between the content of teaching, the more stressed they were. This result may have been due to the specific learning environment during online education. In this learning format, studying may have been more difficult for those students using cognitive strategies, in this case the strategy to connect the learned concepts and theories with existing knowledge. Especially for students who have difficulties to connect the learning content and to transfer their knowledge to other areas on their own, digital education may be a hurdle, as fewer interactions among the students took place [31]. Medical students from Austria have noted that face-to-face learning is better for a shared understanding of the learning content [32]. However, also several other mediating variables are conceivable here. For example, communication with the lecturer that may have been less frequent and mainly via non-personal communication channels (e.g., E-Mail) or other ways of acquiring the learning content (e.g., reading texts or asynchronous online lectures) could have influenced this relationship [27]. Even though the use of cognitive learning strategies is associated with academic performance [9] this does not necessarily seem to apply to digital education. This indicates that self-regulated learning in online formats and digital literacy need to be further developed. In addition, the role of motivation in this context would have to be investigated. The question arises whether self-regulation competencies are generally present and students do not have sufficient motivation to engage in a thoughtful process in digital education [33].

Further, success in study tasks was negatively related to cooperative learning in our study. Students who prefer to learn with their peers were less successful in mastering the study tasks during the online semester. While this result is contrary to previous studies, in which cooperative learning was positively associated with academic achievement [34–36], the opposite association in our sample is not surprising for the context of digital education. Cooperative learning is characterized by students helping each other to understand the academic content [37], which is lacking during digital education. Additionally, the mechanisms that are discussed why cooperative learning promotes academic achievement, for instance motivation, social-cohesion, and cognitive aspects [38] are neglected within this learning format. Thus, students who prefer cooperative learning were unable to draw on these resources during online sessions, making it more difficult for them to successful complete the study tasks. This result highlights how absence from class affects effective learning and emphasizes the fact of fostering peer interactions during digital education where possible. This didactic approach is essential in the education of medical students, since communication opportunities and social interactions are important to develop relevant skills for future physicians.

### Strengths and limitations

The present study has some limitations that need to be considered when interpreting our results. First, we used a cross-sectional design and therefore no statements can be made about the causality of the associations examined. Second, we used students’ self-report to investigate study skills and dispositions which may have biased the results. On the other hand, self-reports are the most common way to assess learning strategies and achievement emotions in higher education and we relied on well-established instruments that have been validated on German students. Third, although we only disseminated access to the study via a link through the medical faculty email distribution list, we cannot completely rule out the possibility that individuals who are not part of the study population may have participated. Fourth, it has to be kept in mind that our findings may only apply to students at a medical faculty in Germany. For one thing cultural specificities in learning behavior have been found [39] and for another the study of medicine differs significantly from other study subjects regarding scope of the learning material and the modality of seminars and examinations. Nevertheless, this is one of the few studies examining associations between learners’ characteristic and stress, mastery experiences as well as enjoyment in the context of digital education during the COVID-19 pandemic in a large sample of students. We encourage future researchers to examine the underlying mechanisms that are relevant for enjoyment and success in the case of online teaching. Also, we recommend replication of the current study using a longitudinal design and different study subjects.

### Implications for digital education at medical faculties

Considering the specific situation during digital education, it may be beneficial for students to promote cooperative learning environments. For example, reducing teacher-centred teaching and instead giving students the opportunity to discuss the study materials in breakout sessions can do this. Further, those discussions may promote deep-level learning, like establishing associations, which in turn helps to built-up self-efficacy beliefs that are known to foster successful learning and academic achievement [8, 9, 40]. Because many students have indicated that they miss their fellow students, lecturers should also make it possible that students are given space to communicate and foster cohesion beyond the content of the lecture in those breakout sessions. In addition, the training of metacognitive learning strategies could help to ensure that students enjoy online teaching more and are better in organizing their studies. Thereby, improvements in metacognition are to be expected particularly when concrete feedback is received from the teacher [41]. In order to cope with test anxiety and reduce stress experiences, teachers should help their students to understand and manage the physio-affective and cognitive symptoms. For this, cognitive-behavioral interventions, e.g. relaxation or examination simulations during online sessions, may be useful strategies. In any case, lecturers should always be aware that students have different ways of dealing with digital education and thus differ in learning success wherefore some may need special support. Differences in motivations and expectations toward online learning and digital competence in particular must be taken into account [42].

## Data Availability

All data and supplementary material are available upon request from the corresponding author.

## References

[1] Pather N, Blyth P, Chapman JA, Dayal MR, Flack NAMS, Fogg QA (2020). Forced disruption of anatomy education in Australia and New Zealand: an acute response to the Covid-19 pandemic. Anat Sci Educ.

[2] Sandhu P, Wolf M de. The impact of COVID-19 on the undergraduate medical curriculum. Med Educ Online 2020;25:1764740. 10.1080/10872981.2020.1764740. 10.1080/10872981.2020.1764740 PMC7269089 32400298

[3] Bernard RM, Abrami PC, Lou Y, Borokhovski E, Wade A, Wozney L (2004). How does distance education compare with classroom instruction?: a Meta-analysis of the empirical literature. Rev Educ Res.

[4] Puthran R, Zhang MWB, Tam WW, Ho RC (2016). Prevalence of depression amongst medical students: a meta-analysis. Med Educ.

[5] La Fuente J d, López M, Zapata L, Martínez-Vicente JM, Vera MM, Solinas G, Fadda S (2014). Competency to study and learn in stressful contexts: fundamentals of the "e-coping with academic stress" utility. Electron J Res Educ Psychol.

[6] La Fuente J d, Amate J, Sander P (2018). Relationships between cognitive strategies, motivational strategies and academic stress in professional examination candidates. Electron J Res Educ Psychol.

[7] Roick J, Ringeisen T (2017). Self-efficacy, test anxiety, and academic success: a longitudinal validation. Int J Educ Res.

[8] Roick J, Ringeisen T (2018). Students' math performance in higher education: examining the role of self-regulated learning and self-efficacy. Learn Individ Differ.

[9] Richardson M, Abraham C, Bond R (2012). Psychological correlates of university students' academic performance: a systematic review and meta-analysis. Psychol Bull.

[10] Pajares F (1996). Self-efficacy beliefs in academic settings. Rev Educ Res.

[11] Pekrun R, Elliot AJ, Maier MA (2009). Achievement goals and achievement emotions: testing a model of their joint relations with academic performance. J Educ Psychol.

[12] Boekaerts M, Pintrich PR, Zeidner M (2005). Handbook of self-regulation.

[13] Boerner S, Seeber G, Keller H, Beinborn P (2005). Lernstrategien und Lernerfolg im Studium [learning strategies and success in higher education]. Zeitschrift Entwicklungspsychol Pädagogische Psychol.

[14] Robbins SB, Lauver K, Le H, Davis D, Langley R, Carlstrom A (2004). Do psychosocial and study skill factors predict college outcomes? A meta-analysis. Psychol Bull.

[15] Ghaith G (2002). The relationship between cooperative learning, perception of social support, and academic achievement. System..

[16] Jerusalem M, Satow L, Schwarzer R, Jerusalem M (1999). Schulbezogene Selbstwirksamkeitserwartung [academic self-efficacy]. Skalen zur Erfassung von Lehrer-und Schülermerkmalen.

[17] Raufelder D, Ringeisen T (2016). Self-perceived competence and test anxiety: the role of academic self-concept and self-efficacy. J Individ Differ.

[18] Hodapp V, Rohrmann S, Ringeisen T (2011). Prüfungsangstfragebogen. [Test Anxiety Questionnaire].

[19] Hoferichter F, Raufelder D, Ringeisen T, Rohrmann S, Bukowski WM (2016). Assessing the multi-faceted nature of test anxiety among secondary school students: an English version of the German test anxiety questionnaire: PAF-E. J Psychol.

[20] Ringeisen T, Raufelder D, Schnell K, Rohrmann S (2016). Validating the proposed structure of the relationships among test anxiety and its predictors based on control-value theory: evidence for gender-specific patterns. Educ Psychol.

[21] Nelson Laird TF, Shoup R, Kuh GD, Schwarz MJ (2008). The effects of discipline on deep approaches to student learning and college outcomes. Res High Educ.

[22] Marsh HW, Hau K-T, Artelt C, Baumert J, Peschar JL (2006). OECD's brief self-report measure of Educational Psychology's Most useful affective constructs: cross-cultural, psychometric comparisons across 25 countries. Int J Test.

[23] Owens L, Straton RG (1980). The development of a co-operative, competitive, and individualised learning preference scale for students. Br J Educ Psychol.

[24] Verma JP (2013). Data analysis in management with SPSS software.

[25] Rubin DB (1987). Multiple imputation for nonresponse in surveys.

[26] Tuma F, Nassar AK, Kamel MK, Knowlton LM, Jawad NK (2021). Students and faculty perception of distance medical education outcomes in resource-constrained system during COVID-19 pandemic. A cross-sectional study. Ann Med Surg (Lond)..

[27] Sindiani AM, Obeidat N, Alshdaifat E, Elsalem L, Alwani MM, Rawashdeh H (2020). Distance education during the COVID-19 outbreak: a cross-sectional study among medical students in north of Jordan. Ann Med Surg (Lond).

[28] Putwain D, Sander P, Larkin D (2013). Academic self-efficacy in study-related skills and behaviours: relations with learning-related emotions and academic success. Br J Educ Psychol.

[29] Pajares F, Valiante G (2001). Gender differences in writing motivation and achievement of middle school students: a function of gender orientation?. Contemp Educ Psychol.

[30] Pajares F, Britner SL (2001). Self-efficacy beliefs, motivation, race, and gender in middle school science. J Women Minor Scien Eng.

[31] Al-Balas M, Al-Balas HI, Jaber HM, Obeidat K, Al-Balas H, Aborajooh EA (2020). Distance learning in clinical medical education amid COVID-19 pandemic in Jordan: current situation, challenges, and perspectives. BMC Med Educ.

[32] Paechter M, Maier B (2010). Online or face-to-face?: Students' experiences and preferences in e-learning. Internet High Educ.

[33] Vissers D, Rowe M, Islam MS, Taeymans J (2018). Ownership and attitudes towards technology use in physiotherapy students from seven countries. Health Prof Educ.

[34] McMaster KN, Fuchs D (2002). Effects of cooperative learning on the academic achievement of students with learning disabilities: an update of Tateyama-Sniezek's review. Learn Disabil Res Pract.

[35] Vaughan W (2002). Effects of cooperative learning on achievement and attitude among students of color. J Educ Res.

[36] Gull F, Shehzad S (2015). Effects of cooperative learning on students’ academic achievement. EduLearn..

[37] Slavin RE. Cooperative learning and academic achievement: why does Groupwork work? [Aprendizaje cooperativo y rendimiento académico: ¿por qué funciona el trabajo en grupo?]. Analesps. 2014. 10.6018/analesps.30.3.201201.

[38] Salvin R, Reynolds W, Miller G, Weiner I (2013). Cooperative learning and achievement: theory and research. Handbook of psychology.

[39] Roth A, Ogrin S, Schmitz B (2016). Assessing self-regulated learning in higher education: a systematic literature review of self-report instruments. Educ Asse Eval Acc.

[40] Schmitz B, Wiese BS (2006). New perspectives for the evaluation of training sessions in self-regulated learning: time-series analyses of diary data. Contemp Educ Psychol.

[41] Miller TM, Geraci L (2011). Training metacognition in the classroom: the influence of incentives and feedback on exam predictions. Metacognition Learn.

[42] Díaz-Noguera MD, Hervás-Gómez C, La Calle-Cabrera AM, de, López-Meneses E. Autonomy, motivation, and digital pedagogy are key factors in the perceptions of Spanish higher-education students toward online learning during the COVID-19 pandemic. Int J Environ Res Public Health. 2022. 10.3390/ijerph19020654. 10.3390/ijerph19020654 PMC8776181 35055475

